# Co-expression of AFAP1-AS1 and PD-1 predicts poor prognosis in nasopharyngeal carcinoma

**DOI:** 10.18632/oncotarget.16545

**Published:** 2017-03-24

**Authors:** Yanyan Tang, Yi He, Lei Shi, Liting Yang, Jinpeng Wang, Yu Lian, Chunmei Fan, Ping Zhang, Can Guo, Shanshan Zhang, Zhaojian Gong, Xiayu Li, Fang Xiong, Xiaoling Li, Yong Li, Guiyuan Li, Wei Xiong, Zhaoyang Zeng

**Affiliations:** ^1^ The Key Laboratory of Carcinogenesis of the Chinese Ministry of Health, Xiangya Hospital, Central South University, Changsha, Hunan, China; ^2^ The Key Laboratory of Carcinogenesis and Cancer Invasion of the Chinese Ministry of Education, Cancer Research Institute, Central South University, Changsha, Hunan, China; ^3^ Hunan Key Laboratory of Nonresolving Inflammation and Cancer, Disease Genome Research Center, The Third Xiangya Hospital, Central South University, Changsha, Hunan, China; ^4^ Hunan Key Laboratory of Translational Radiation Oncology, Hunan Cancer Hospital and The Affiliated Cancer Hospital of Xiangya School of Medicine, Central South University, Changsha, Hunan, China; ^5^ Department of pathology, The Second Xiangya Hospital, Central South University, Changsha, Hunan, China; ^6^ School of Information Science and Engineering, Central South University, Changsha, Hunan, China; ^7^ Department of Cancer Biology, Lerner Research Institute, Cleveland Clinic, Cleveland, Ohio, USA

**Keywords:** long non-coding RNA, *AFAP1-AS1*, programmed death 1 (*PD-1*), prognosis, nasopharyngeal carcinoma (NPC)

## Abstract

Nasopharyngeal carcinoma (NPC) carries a high potential for metastasis and immune escape, with a great risk of relapse after primary treatment. Through analysis of whole genome expression profiling data in NPC samples, we found that the expression of a long non-coding RNA (lncRNA), actin filament-associated protein 1 antisense RNA 1 (*AFAP1-AS1*), is significantly correlated with the immune escape marker programmed death 1 (*PD-1*). We therefore assessed the expression of *AFAP1-AS1* and *PD-1* in a cohort of 96 paraffin-embedded NPC samples and confirmed that *AFAP1-AS1* and *PD-1* are co-expressed in infiltrating lymphocytes in NPC tissue. Moreover, patients with high expression of *AFAP1-AS1* or *PD-1* in infiltrating lymphocytes were more prone to distant metastasis, and NPC patients with positive expression of both *AFAP1-AS1* and *PD-1* had the poorest prognosis. This study suggests that *AFAP1-AS1* and *PD-1* may be potential therapeutic targets in NPC and that patients with co-expression of *AFAP1-AS1* and *PD-1* may be ideal candidates for future clinical trials of anti-PD-1 immune therapy.

## INTRODUCTION

Long non-coding RNAs (lncRNAs) are a group of RNA transcripts that exceed 200 nt in length yet lack significant open reading frames (ORFs) [1]. They regulate gene expression through transcriptional, post-transcriptional and epigenetic effects [2–8]. Tens of thousands of lncRNAs have been identified in the human genome [9], many of which are abnormally expressed in a variety of human tumors, and are involved in various stages of carcinogenesis, including tumor initiation, progression and metastasis [10–17]. However, the function of the vast majority of these lncRNAs is still unclear.

In a previous study, we performed gene expression profile (GEP) analysis by microarray and found that one lncRNA named actin filament-associated protein 1 antisense RNA1 (*AFAP1-AS1*) was significantly upregulated in nasopharyngeal carcinoma (NPC), and promoted invasion and metastasis of cancer cells by regulating the expression of several small GTPase family members and molecules in the actin cytokeratin signaling pathway [18]. However, it is not yet known whether there is any other biological function of *AFAP1-AS1* in the tumorigenesis of NPC.

In this study, using GEP dataset, we found that a key molecular maker of tumor immune evasion, programmed death 1 (*PD-1*), was positively correlated with the expression of *AFAP1-AS1*. Therefore, we used *in situ* hybridization to detect the expression of *AFAP1-AS1* and immunohistochemical staining to detect the expression of *PD-1* in a cohort of 96 NPC biopsies, and we analyzed co-expression of *AFAP1-AS1* and *PD-1* and its relevance in clinical outcomes and prognosis. The results suggest that *AFAP1-AS1* might be involved in the *PD-1* immune checkpoint pathway and that *PD-1* and *AFAP1-AS1* might jointly promote the formation and development of NPC.

## RESULTS

### The expression of *AFAP1-AS1* is positively correlated with *PD-1* in NPC

**Figure 1:**
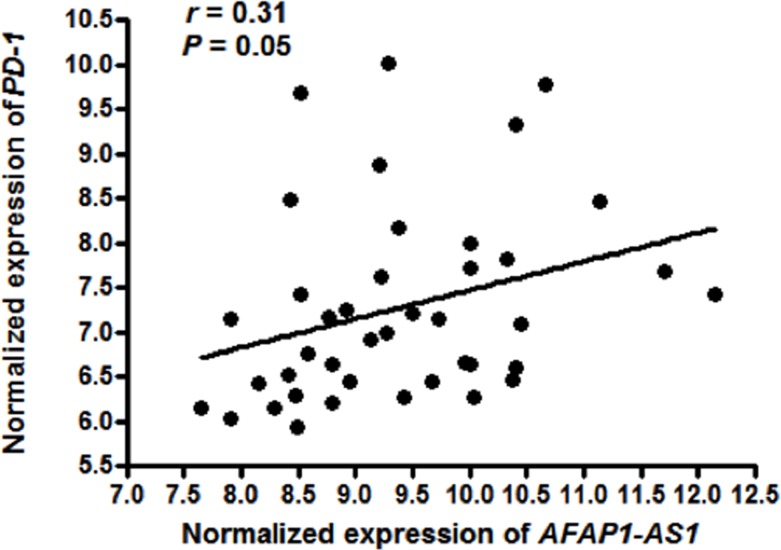
Expression of *AFAP1-AS1* is positively correlated with *PD-1* Normalized gene expression signatures of *AFAP1-AS1* and *PD-1* were derived from whole-genome GEP dataset GSE12452. Expression of *AFAP1-AS1* and *PD-1* was positively correlated in 31 NPC samples and 10 NPE samples (*r*=0.31, *P*=0.05).

The Gene Expression Omnibus (GEO) database [19] is a public gene expression data repository that serves as a valuable data repository for biomedical research and has collected a large amount of gene expression data for data mining [20]. Mining of published high-throughput data is a commonly used and low-cost method for identifying novel biomarkers and gaining insight into the biological functions of novel genes [21–23]. To identify potential novel functions of *AFAP1-AS1*, we downloaded a GEP dataset, GSE12452, from the GEO database; this dataset consists of 41 samples of whole-genome GEP data, including 10 samples of non-tumor nasopharyngeal epithelial (NPE) biopsies and 31 cases of NPC [24]. We found that there were 4196 differentially expressed genes in the GSE12452 dataset. Among these differentially expressed genes, *AFAP1-AS1* was highly expressed in NPC cells and was positively correlated with the expression of *PD-1*, a key molecular marker of tumor immune evasion (Figure 1, *P*=0.05).

### *AFAP1-AS1* and *PD-1* are co-expressed in infiltrating lymphocytes in NPC tissue

**Figure 2:**
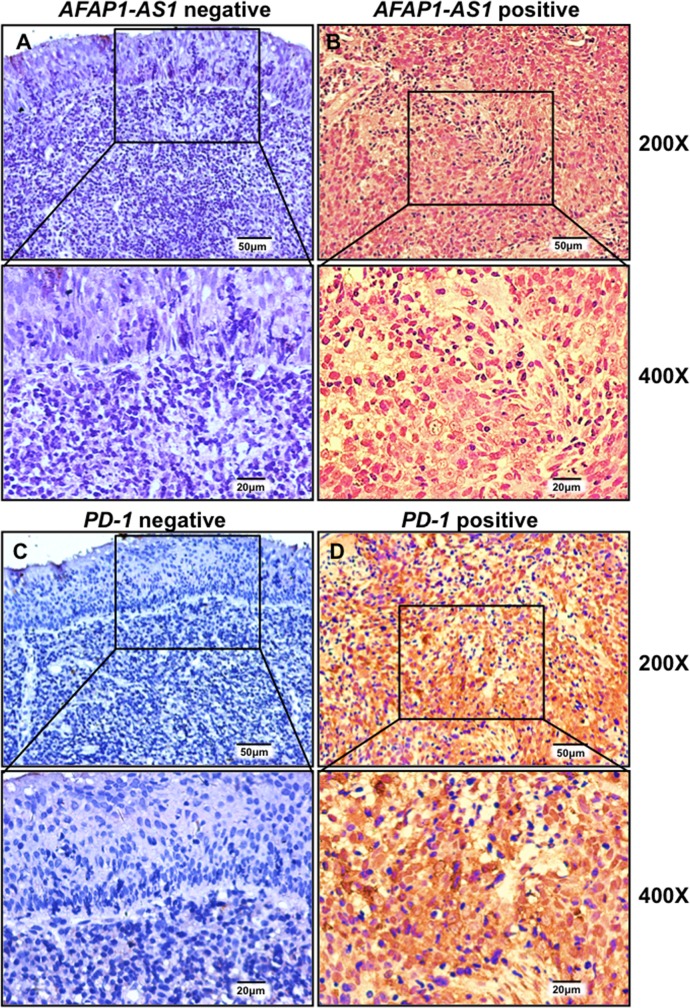
*AFAP1-AS1* and *PD-1* are highly and jointly expressed in infiltrating lymphocytes of NPC tissues Representative images of *in situ* hybridization for *AFAP1-AS1* **(A & B)** and immunohistochemical staining for *PD-1* **(C & D)** are shown. *AFAP1-AS1* and *PD-1* are negatively expressed in adjacent non-tumor NPE tissue (A & C) but highly expressed in infiltrating lymphocytes (B & D).

Since *PD-1* is a membrane protein and mainly expressed on the lymphocyte cell surface [25]; and tumor-infiltrating lymphocytes are associated with the development and progression of NPC [26]. we set out to assess *AFAP1-AS1* and *PD-1* expression in a cohort of 96 paraffin-embedded NPC samples *via in situ* hybridization and immunohistochemical staining, respectively (Supplementary Table 1). Expression of *AFAP1-AS1* was absent or very low in adjacent non-tumor NPE (Figure 2A) but high in NPC cells and infiltrating lymphocytes in 68 of 96 cases (70.8%, Figure 2B). Similarly, the expression of *PD-1* was low or negative in non-tumor NPE (Figure 2C) but high in infiltrating lymphocytes surrounding NPC cells (36 of 96 cases, 37.5%, Figure 2D).

### High expression of *AFAP1-AS1* or *PD-1* is correlated with distant metastasis at relapse

**Figure 3:**
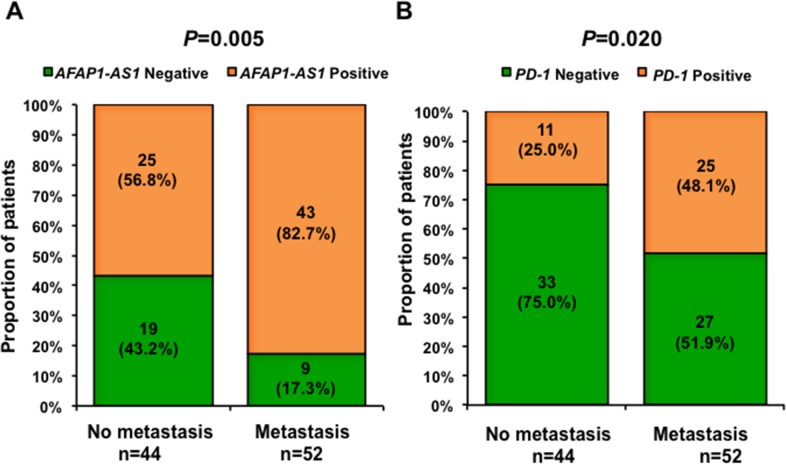
Expression of *AFAP1-AS1* or *PD-1* is associated with distant metastasis Patients with high expression of *AFAP1-AS1* **(A)** or *PD-1* **(B)** in NPC tumor-infiltrating lymphocytes were more likely to have distant metastasis when they relapsed.

We then analyzed the correlation between the expression of *AFAP1-AS1* and *PD-1* and clinicopathological features of these 96 NPC patients. There was no significant correlation between the expression of *AFAP1-AS1* or *PD-1* and patients' gender, age at diagnosis, tumor size (T stage), lymphatic invasion (N stage), distant metastasis at diagnosis (M stage) and overall clinical staging (Supplementary Table 2), but patients with high expression of *AFAP1-AS1* or *PD-1* in NPC-infiltrating lymphocytes were more likely to have distant metastasis when they relapsed (Figure 3A and 3B, P=0.005 and *P*=0.020, respectively).

### Co-expression of *AFAP1-AS1* and *PD-1* predicts poor prognosis of NPC

**Figure 4:**
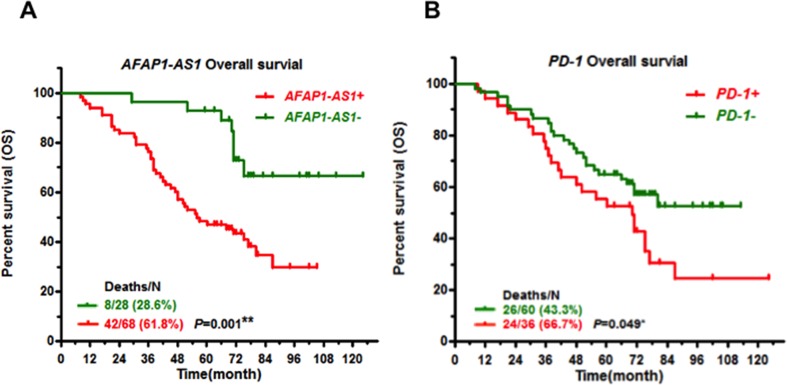
High expression of *AFAP1-AS1* or PD-1 predicts poor prognosis Kaplan-Meier survival curves of patients with NPC, stratified by *AFAP1-AS1* expression **(A)** and *PD-1* expression **(B)**, shows that high expression of *AFAP1-AS1* or *PD-1* predicts poor prognosis.

**Figure 5:**
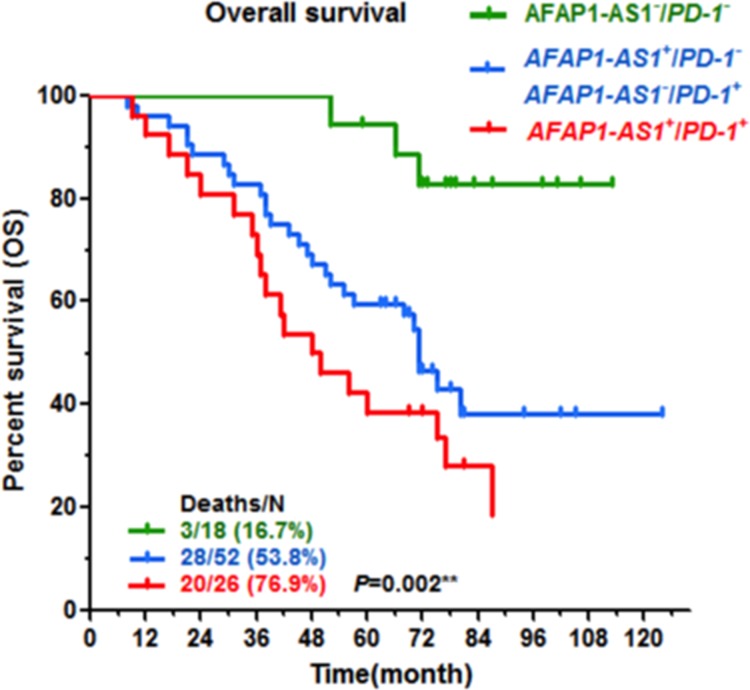
Co-expression of *AFAP1-AS1* and *PD-1* predicts the poorest outcomes Patients with high expression of both *AFAP1-AS1* and *PD-1* had a significantly less favorable prognosis than those with low expression of *AFAP1-AS1* and/or *PD-1*.

Finally, we analyzed the association of *AFAP1-AS1* and *PD-1* expression with NPC patients' outcomes. Patients with positive expression of *AFAP1-AS1* or *PD-1* in NPC biopsies had a poor prognosis, with shorter overall survival (47.7% five-year survival with positive expression of *AFAP1-AS1* vs. 92.8% with negative expression, *P*=0.001, Figure 4A; 52.8% five-year survival with positive *PD-1* expression vs. 65.0% with negative expression, *P*=0.049, Figure 4B). NPC patients with positive expression of both *AFAP1-AS1* and *PD-1* had much shorter overall survival (38.5% five-year survival, *P*=0.002, Figure 5).

## DISCUSSION

NPC is an Epstein-Barr Virus (EBV)-associated malignancy and is the most common malignant head and neck tumor, originating in the nasopharyngeal epithelium [27–31]. High incidences of NPC are observed in Southeast Asia and southern China, resulting in serious healthcare problems in these regions [32–34]. NPC is highly metastatic and heterogeneous compared with other head and neck tumors [35–40]. Radiotherapy has been used as the primary clinical treatment for all stages of NPC over the past several decades, but many patients eventually die due to recurrence and distant metastasis [41–43]. Although recent studies have shown that induction chemotherapy plus concurrent chemoradiotherapy significantly improves failure-free survival in locally or regionally advanced NPC with acceptable toxicity, long-term efficacy and toxicities remain unclear [44]. Management of advanced NPC is therefore a highly challenging issue, and novel and effective therapies for NPC are urgently needed.

Recently, tumor immune evasion has emerged as a hallmark of cancer progression [45–47]. Immune surveillance is an important mechanism in preventing the development of cancer and inhibiting tumor growth and metastasis [48–50]. There are many immunosuppressive mechanisms in the tumor microenvironment that can decrease the activity of tumor-infiltrating lymphocytes and increase the risk of tumor metastasis and recurrence [51]. Among these mechanisms, T cell-mediated immune responses, especially CD8^+^ cytotoxic T lymphocytes, play an important role in tumor immunity [52, 53].

PD-1 is a transmembrane receptor that is mainly expressed on T cells. It was first cloned in T cell hybridomas and was named “programmed death receptor” because of its involvement in T cell apoptosis [25]. In tumor tissues, *PD-1* is mainly expressed in tumor-infiltrating lymphocytes (TILs) [54, 55]. High expression of PD-1 in TILs led to depletion and deactivation of T cells [56–60]. PD-1 also interacts with programmed death ligand-1 (PD-L1) and programmed death ligand-2 (PD-L2), which are mainly expressed on the surface of tumor cells or in the tumor matrix [61, 62]; these ligands activate PD-1, which then inhibits the proliferation of T cells and promotes the immune escape of tumor cells, playing an important role in immune suppression and cancer progression [63–65]. The blockade of immune checkpoints has been the most promising approach to activating antitumor immunity. A tumor immunotherapy treatment strategy using a combined PD-1/PD-L1 antibody has entered the stage of clinical trials and shown good performance [66, 67].

It has been reported that local infiltration of T cells is a favorable indicator of survival in NPC patients, but many studies have indicated that NPC can escape immune surveillance through various mechanisms [68–75]. Recent studies have shown that NPC has high levels of PD-L1 and PD-1, indicating that NPC may be a candidate for PD-1/PD-L1-dericted therapies [76–78]. However, the underlying mechanism of PD-1 regulation in NPC is undetermined.

In a previous study, we found that the lncRNA *AFAP1-AS1* is significantly upregulated in NPC and promotes invasion and metastasis of cancer cells [18]. Interestingly, using the GEO database, we found and confirmed that the expression of *AFAP1-AS1* is positively correlated with *PD-1*, that high expression of *PD-1* and *AFAP1-AS1* predicts high incidence of recurrence or metastasis and that co-expression of *AFAP1-AS1* and *PD-1* in NPC biopsies predicted the poorest prognosis.

However, there are still several relevant mechanism-related questions to be solved urgently. For example, is there a regulatory relationship between *AFAP1-AS1* and *PD-1*? Does *AFAP1-AS1* promote the expression of *PD-1*? And how does *AFAP1-AS1* regulate *PD-1*? We speculate that *AFAP1-AS1* may regulate *PD-1* expression through the following mechanisms. First, *AFAP1-AS1* may act as a competing endogenous RNA (ceRNAs) [79–81] to regulate *PD-1* expression. Second, *AFAP1-AS1* may bind to certain transcriptional complexes to regulate *PD-1* transcription. Third, *AFAP1-AS1* may affect epigenetic modification of *PD-1*. These questions warrant in-depth exploration in future studies.

In conclusion, to our knowledge, this is the first study to explore the co- expression of a lncRNA, *AFAP1-AS1*, and an immune escape marker, *PD-1*, in tumor-infiltrating lymphocytes among NPC patients, as well as their synergistic effect on prognosis. This study provides two potential therapeutic targets for NPC, *AFAP1-AS1* and *PD-1*, to inhibit tumor metastasis and stimulate anti-tumor immunity. Patients with higher expression of both *AFAP1-AS1* and *PD-1* might be ideal candidates for future clinical trials of anti-PD-1 therapy. Our study is limited by its retrospective nature, with a relatively small sample size. Further studies with larger sample sizes are warranted.

## MATERIALS AND METHODS

### Tissue samples

A total of 96 samples of paraffin-embedded NPC tissue were collected from newly diagnosed NPC patients at the Xiangya Hospital and the Affiliated Cancer Hospital of Central South University (Changsha China). All specimens were confirmed by histopathological examination. All of the patients had received routine radiotherapy. This study was approved by the Research Ethics Board of Xiangya Hospital and the Affiliated Cancer Hospital of Central South University, and signed informed consent was obtained from each participant before they were enrolled in the study. Clinicopathological data were collected from patient medical records and are reported in Supplementary Table 1.

### *In situ* hybridization

*In situ* hybridization was performed to detect the expression of *AFAP1-AS1* in tissue specimens using three 30-nucleotide probes from different regions of *AFAP1-AS1*. GAPDH was used as a positive control. The probe sequences were as follows.

AFAP1-AS1 probes:

Probe 1: 5′- ATTCCTTTATTTTATGGGATGTTCTGTAGGGAGTT-3′,

Probe 2: 5′-TAGAAATGAGAAAAGAATCACCAAGAGAGTAAGCA -3′,

Probe 3: 5′-CCCTACAGCTAGTTTCCTCTTCATTTATTCATTT-3′

*GAPDH* probes:

Probe 1: 5′-CCACTTTACCAGAGTTAAAAGCAGCCCTGG-3′

Probe 2: 5′-CAGTAGAGGCAGGGATGATGTTCTGGAGAG-3′

Probe 3: 5′-GTCAGAG GAGACCACCTGGTG CTCAGTGTA-3′

The probes were synthesized and labeled with DIG-dUTP at the 3′ end using a kit from Invitrogen (Shanghai, China) [82–84]. The *in situ* hybridization results were independently scored manually by two pathologists who counted 20 sequential high-power fields judged to be representative of the tumor, while remaining blinded to clinical information.

### Immunohistochemistry

Paraffin-embedded sections (3 μm) were used for PD-1 staining. Paraffin sections were dewaxed using turpentine and gradient alcohol, immersed in 3% H_2_O_2_ at room temperature for 10 min and then treated with citric acid buffer [85, 86]. Staining for *PD-1* (Proteintech, Wuhan, China) was observed under the microscope. Samples were divided into a *PD-1*-negative group and a *PD-1*-positive group by double-blind scoring by two pathologists.

### Data analysis

We downloaded an NPC gene expression dataset from the GEO database (accession number GSE12452). The GSE12452 microarray consists of 10 non-tumor NPE biopsies and 31 cases of NPC [24]. We used Significant Analysis of Microarray (SAM) software [87] to analyze the microarray expression profiles(cut-off=1.5, FDR<0.05 lncRNA expression) and selected differentially expressed molecules of interest for the subsequent Pearson correlation analysis.

Pearson correlation analysis was used to evaluate the expression levels of *AFAP1-AS1* and *PD-1*. The Chi-squared test was used to evaluate the expression of *AFAP1-AS1*, *PD-1* and clinicopathological features such as gender, age at diagnosis, TNM staging, and metastasis, among others. Survival analysis was performed using the Kaplan-Meier test. A threshold of *P*<0.05 was used to indicate statistical significance, and all tested *P* values were two-sided. Statistical analysis was performed using SPSS 13 and GraphPad 5 software

## SUPPLEMENTARY TABLES







## Abbreviations

NPC: nasopharyngeal carcinoma
LncRNAs: Long noncoding RNAs
*AFAP1-AS1*: actin lament associated protein 1 antisense RNA1
*PD-1*: programmed death 1
ORF: open reading frame
GEP: gene expression profile
GEO: Gene Expression Omnibus
NPE: nasopharyngeal epithelial
EBV: Epstein-Barr Virus
TILs: tumor infiltrating lymphocytes
*PD-L1*: programmed death ligand-1
*PD-L2*: programmed death ligand-2
ceRNAs: Competing Endogenous RNAs
SAM: Significant Analysis of Microarray.
