# Supplementary material for: Co-expression of AFAP1-AS1 and PD-1 predicts poor prognosis in nasopharyngeal carcinoma
Source: Oncotarget. 2017 Mar 24;8(24):39001–11. doi: 10.18632/oncotarget.16545 (PMC5503590; doi:10.18632/oncotarget.16545)
Supplement: Supplementary file 1 [file oncotarget-08-39001-s001.pdf]

Co-expression of AFAP1-AS1 and PD-1 predicts poor prognosis in nasopharyngeal carcinoma

SUPPLEMENTARY TABLES

Supplementary Table 1: Clinicopathological data and expression levels of *AFAP1-AS1* and *PD-1*.

Supplementary File 1

Supplementary Table 2: Correlation of clinical parameters with *AFAP1-AS1* and *PD-1* in nasopharyngeal carcinoma.

Supplementary File 2
