# Supplementary material for: Co-expression of AFAP1-AS1 and PD-1 predicts poor prognosis in nasopharyngeal carcinoma
Source: Oncotarget. 2017 Mar 24;8(24):39001–11. doi: 10.18632/oncotarget.16545 (PMC5503590; doi:10.18632/oncotarget.16545)
Supplement: Supplementary file 2 [file oncotarget-08-39001-s002.doc]

**Supplemental Table S1.** Clinicopathological data and expression levels of *AFAP1-AS1* and *PD-1*

| Patient No.1 | Gender (M=Male F=Female) | Age at Diagnosis | T stage | N stage | M stage | Clinic stages | Relapse status | *PD1* expression level(1=positive,0=negtive) | *AFAP1-AS1* expression level(1=positive,0=negtive) | Over-all survival (OS) status (0=alive, 1=death) | OS time (Months) |
| --- | --- | --- | --- | --- | --- | --- | --- | --- | --- | --- | --- |
| Pat 039 | M | 23 | 3 | 2 | 0 | III | Metastasis | 0 | 1 | 1 | 37 |
| Pat 088 | M | 24 | 4 | 0 | 0 | IVa |  | 0 | 1 | 0 | 70 |
| Pat 091 | M | 27 | 3 | 1 | 0 | III | Metastasis | 0 | 1 | 1 | 8 |
| Pat 092 | M | 27 | 3 | 2 | 0 | III | Metastasis | 0 | 1 | 1 | 10 |
| Pat 055 | M | 28 | 4 | 2 | 0 | IVa | Metastasis | 1 | 1 | 1 | 87 |
| Pat 089 | M | 29 | 2 | 2 | 0 | III | Metastasis | 0 | 1 | 0 | 105 |
| Pat 004 | M | 29 | 2 | 3 | 0 | IVa | Metastasis | 1 | 0 | 1 | 70 |
| Pat 073 | M | 31 | 2 | 2 | 0 | III | Metastasis | 1 | 1 | 1 | 50 |
| Pat 067 | M | 32 | 2 | 2 | 0 | III | Metastasis | 0 | 1 | 1 | 39 |
| Pat 029 | M | 32 | 2 | 3 | 0 | IVa |  | 0 | 0 | 0 | 73 |
| Pat 077 | F | 33 | 2 | 0 | 0 | II | Metastasis | 1 | 1 | 0 | 87 |
| Pat 047 | F | 34 | 3 | 1 | 0 | III | Metastasis | 0 | 1 | 1 | 48 |
| Pat 035 | M | 35 | 2 | 1 | 0 | II | Metastasis | 0 | 1 | 1 | 45 |
| Pat 104 | M | 35 | 2 | 2 | 0 | III | Metastasis | 0 | 1 | 1 | 22 |
| Pat 063 | F | 35 | 3 | 1 | 0 | III |  | 0 | 1 | 0 | 75 |
| Pat 008 | M | 35 | 3 | 2 | 0 | III | In situ relapse | 0 | 0 | 0 | 101 |
| Pat 019 | M | 36 | 2 | 1 | 0 | II | In situ relapse | 0 | 0 | 0 | 98 |
| Pat 076 | F | 36 | 2 | 2 | 0 | III | Metastasis | 0 | 1 | 0 | 102 |
| Pat 069 | M | 37 | 1 | 1 | 0 | II | Metastasis | 0 | 1 | 1 | 47 |
| Pat 068 | M | 37 | 2 | 2 | 0 | III | Metastasis | 0 | 1 | 1 | 55 |
| Pat 061 | M | 37 | 2 | 2 | 0 | III | Metastasis | 1 | 1 | 1 | 77 |
| Pat 107 | F | 38 | 2 | 1 | 0 | II | Metastasis | 0 | 1 | 1 | 43 |
| Pat 024 | M | 38 | 2 | 2 | 0 | III |  | 0 | 0 | 0 | 73 |
| Pat 110 | F | 38 | 2 | 2 | 0 | III |  | 0 | 1 | 0 | 78 |
| Pat 072 | M | 39 | 1 | 1 | 0 | II | Metastasis | 1 | 1 | 1 | 36 |
| Pat 038 | M | 39 | 1 | 2 | 0 | III | Metastasis | 1 | 1 | 1 | 38 |
| Pat 071 | M | 39 | 2 | 2 | 0 | III | Metastasis | 1 | 1 | 1 | 56 |
| Pat 027 | M | 39 | 2 | 2 | 0 | III |  | 0 | 0 | 0 | 78 |
| Pat 079 | M | 39 | 2 | 2 | 0 | III | Metastasis | 1 | 1 | 0 | 87 |
| Pat 022 | M | 39 | 2 | 3 | 0 | IVa |  | 1 | 0 | 0 | 124 |
| Pat 075 | M | 39 | 2 | 3 | 0 | IVa | In situ relapse | 0 | 1 | 1 | 71 |
| Pat 020 | M | 40 | 2 | 1 | 0 | II | In situ relapse | 0 | 0 | 0 | 113 |
| Pat 105 | M | 40 | 3 | 1 | 0 | III | Metastasis | 0 | 1 | 1 | 30 |
| Pat 051 | F | 41 | 2 | 1 | 0 | II | In situ relapse | 0 | 1 | 0 | 81 |
| Pat 101 | M | 41 | 2 | 2 | 0 | III | Metastasis | 1 | 1 | 1 | 24 |
| Pat 030 | M | 41 | 2 | 2 | 0 | III |  | 0 | 0 | 0 | 83 |
| Pat 056 | M | 42 | 1 | 0 | 0 | I |  | 1 | 1 | 0 | 69 |
| Pat 060 | M | 42 | 1 | 2 | 0 | III | Metastasis | 0 | 1 | 0 | 94 |
| Pat 014 | F | 42 | 2 | 1 | 0 | II | In situ relapse | 0 | 0 | 0 | 73 |
| Pat 026 | M | 42 | 2 | 2 | 0 | III |  | 1 | 0 | 0 | 71 |
| Pat 062 | M | 42 | 2 | 2 | 0 | III | In situ relapse | 0 | 1 | 0 | 80 |
| Pat 057 | M | 42 | 2 | 3 | 0 | IVa |  | 0 | 1 | 0 | 63 |
| Pat 002 | M | 43 | 1 | 0 | 0 | I | In situ relapse | 0 | 0 | 0 | 87 |
| Pat 065 | F | 43 | 3 | 2 | 0 | III | In situ relapse | 0 | 1 | 0 | 68 |
| Pat 066 | M | 43 | 4 | 1 | 0 | IVa | Metastasis | 0 | 1 | 1 | 51 |
| Pat 040 | M | 44 | 2 | 0 | 0 | II | Metastasis | 0 | 1 | 0 | 102 |
| Pat 111 | F | 44 | 2 | 2 | 0 | III |  | 0 | 1 | 0 | 71 |
| Pat 046 | M | 44 | 3 | 1 | 0 | III | Metastasis | 1 | 1 | 1 | 42 |
| Pat 070 | M | 45 | 2 | 2 | 0 | III | In situ relapse | 0 | 1 | 1 | 38 |
| Pat 043 | M | 45 | 3 | 1 | 0 | III | In situ relapse | 0 | 1 | 1 | 68 |
| Pat 013 | M | 46 | 2 | 1 | 0 | II | In situ relapse | 0 | 0 | 0 | 106 |
| Pat 064 | M | 47 | 2 | 3 | 0 | IVa | Metastasis | 1 | 1 | 1 | 48 |
| Pat 103 | M | 47 | 4 | 2 | 0 | IVa | Metastasis | 1 | 1 | 0 | 75 |
| Pat 025 | M | 48 | 2 | 2 | 0 | III |  | 0 | 0 | 0 | 77 |
| Pat 112 | M | 48 | 2 | 3 | 0 | IVa | Metastasis | 1 | 1 | 1 | 9 |
| Pat 102 | F | 49 | 2 | 2 | 0 | III | Metastasis | 1 | 1 | 1 | 31 |
| Pat 093 | M | 49 | 2 | 3 | 0 | III | Metastasis | 0 | 1 | 1 | 21 |
| Pat 054 | M | 49 | 3 | 2 | 0 | III | In situ relapse | 1 | 1 | 1 | 75 |
| Pat 007 | M | 50 | 2 | 0 | 0 | II | Metastasis | 0 | 0 | 1 | 52 |
| Pat 003 | M | 50 | 3 | 1 | 0 | III | In situ relapse | 0 | 0 | 1 | 66 |
| Pat 012 | F | 50 | 3 | 2 | 0 | III | In situ relapse | 0 | 0 | 0 | 72 |
| Pat 009 | F | 50 | 4 | 1 | 0 | IVa | In situ relapse | 1 | 0 | 0 | 69 |
| Pat 032 | M | 51 | 1 | 3 | 0 | IVa | Metastasis | 1 | 0 | 1 | 29 |
| Pat 086 | M | 51 | 4 | 0 | 0 | IVa |  | 0 | 1 | 0 | 75 |
| Pat 028 | M | 52 | 1 | 2 | 0 | III |  | 0 | 0 | 0 | 79 |
| Pat 082 | M | 52 | 2 | 1 | 0 | II |  | 0 | 1 | 0 | 64 |
| Pat 018 | M | 52 | 2 | 2 | 0 | III | Metastasis | 0 | 0 | 0 | 59 |
| Pat 016 | F | 52 | 2 | 2 | 0 | III | Metastasis | 0 | 0 | 1 | 71 |
| Pat 001 | M | 52 | 3 | 2 | 0 | III | In situ relapse | 1 | 0 | 0 | 102 |
| Pat 094 | M | 53 | 1 | 2 | 0 | III | Metastasis | 1 | 1 | 1 | 37 |
| Pat 031 | M | 53 | 2 | 1 | 0 | II | In situ relapse | 1 | 0 | 1 | 71 |
| Pat 045 | M | 53 | 2 | 2 | 0 | III | Metastasis | 1 | 1 | 1 | 35 |
| Pat 050 | M | 53 | 2 | 2 | 0 | III | Metastasis | 0 | 1 | 0 | 69 |
| Pat 099 | M | 54 | 2 | 1 | 0 | II | Metastasis | 0 | 1 | 1 | 17 |
| Pat 033 | M | 54 | 2 | 1 | 0 | II | Metastasis | 0 | 0 | 0 | 73 |
| Pat 058 | M | 54 | 2 | 1 | 0 | II |  | 0 | 1 | 0 | 74 |
| Pat 011 | F | 54 | 3 | 1 | 0 | III | Metastasis | 1 | 0 | 1 | 75 |
| Pat 037 | M | 55 | 3 | 1 | 0 | III | Metastasis | 0 | 1 | 1 | 31 |
| Pat 087 | M | 56 | 1 | 2 | 0 | III |  | 0 | 1 | 0 | 69 |
| Pat 044 | M | 56 | 2 | 3 | 0 | IVa | Metastasis | 0 | 1 | 1 | 57 |
| Pat 042 | F | 56 | 3 | 1 | 0 | III | In situ relapse | 0 | 1 | 1 | 38 |
| Pat 006 | M | 57 | 3 | 2 | 0 | III | Metastasis | 1 | 0 | 0 | 63 |
| Pat 095 | M | 58 | 2 | 2 | 0 | III | Metastasis | 1 | 1 | 1 | 21 |
| Pat 017 | M | 58 | 3 | 0 | 0 | III | Metastasis | 1 | 0 | 1 | 71 |
| Pat 074 | M | 59 | 2 | 1 | 0 | II | In situ relapse | 1 | 1 | 1 | 60 |
| Pat 109 | M | 59 | 2 | 2 | 0 | III | Metastasis | 1 | 1 | 1 | 41 |
| Pat 097 | M | 60 | 1 | 1 | 0 | II | In situ relapse | 0 | 1 | 1 | 21 |
| Pat 098 | M | 60 | 2 | 2 | 0 | III | Metastasis | 1 | 1 | 1 | 17 |
| Pat 084 | M | 61 | 3 | 2 | 0 | III |  | 1 | 1 | 0 | 81 |
| Pat 036 | M | 62 | 1 | 3 | 1 | IVb | Metastasis | 1 | 1 | 1 | 12 |
| Pat 083 | M | 63 | 3 | 1 | 0 | III |  | 1 | 1 | 0 | 72 |
| Pat 085 | M | 64 | 2 | 0 | 0 | II |  | 0 | 1 | 0 | 72 |
| Pat 053 | M | 66 | 2 | 2 | 0 | III | In situ relapse | 0 | 1 | 1 | 80 |
| Pat 081 | M | 68 | 1 | 1 | 0 | II |  | 1 | 1 | 0 | 77 |
| Pat 041 | M | 68 | 2 | 1 | 0 | III | Metastasis | 0 | 1 | 1 | 52 |
| Pat 052 | M | 70 | 2 | 2 | 0 | III | Metastasis | 0 | 1 | 0 | 66 |
