# Supplementary material for: Co-expression of AFAP1-AS1 and PD-1 predicts poor prognosis in nasopharyngeal carcinoma
Source: Oncotarget. 2017 Mar 24;8(24):39001–11. doi: 10.18632/oncotarget.16545 (PMC5503590; doi:10.18632/oncotarget.16545)
Supplement: Supplementary file 3 [file oncotarget-08-39001-s003.doc]

**Supplemental Table S2.** Correlation of clinical parameters with *AFAP1-AS1* and *PD-1* expression in nasopharyngeal carcinoma

| **Items** | **Cases (n=96)** | ***AFAP1-AS1* negative** | ***AFAP1-AS1* positive** | **chi-square** | | ***P* value** | ***PD-1*  negative** | ***PD-1* positive** | **chi-square** | ***P* value** |
| --- | --- | --- | --- | --- | --- | --- | --- | --- | --- | --- |
| Age (years) |  |  |  |  |  | |  |  |  |  |
| ≤45 | 50 | 12 | 38 | 1.35 | 0.246 | | 35 | 15 | 3.21 | 0.073 |
| ＞45 | 46 | 16 | 30 |  |  | | 24 | 22 |  |  |
| Gender |  |  |  |  |  | |  |  |  |  |
| Male | 80 | 23 | 57 | 0.04 | 0.841 | | 47 | 33 | 1.49 | 0.223 |
| Female | 16 | 5 | 11 |  |  | | 12 | 4 |  |  |
| TNM stage |  |  |  |  |  | |  |  |  |  |
| T stage (T1-2) | 69 | 20 | 49 | 0.00 | 0.950 | | 44 | 25 | 0.17 | 0.682 |
| T stage (T3-4) | 27 | 8 | 19 |  |  | | 16 | 11 |  |  |
| N stage (N0-1) | 40 | 12 | 28 | 0.02 | 0.879 | | 29 | 11 | 2.93 | 0.087 |
| N stage (N2-3) | 56 | 16 | 40 |  |  | | 31 | 25 |  |  |
| Stage I, II | 23 | 8 | 15 | 0.46 | 0.497 | | 17 | 6 | 1.68 | 0.195 |
| Stage III, IV | 73 | 20 | 53 |  |  | | 43 | 30 |  |  |
| Metastasis |  |  |  |  |  | |  |  |  |  |
| No | 44 | 19 | 25 | 7.72 | **0.005** | | 33 | 11 | 5.42 | **0.020** |
| Yes | 52 | 9 | 43 |  |  | | 27 | 25 |  |  |
